# Assessing Changes in Reaction Time Following RAMP Warm-Up and Short-Term Repeated Volleyball Specific Exercise in Young Players

**DOI:** 10.3390/s25010125

**Published:** 2024-12-28

**Authors:** Kacper Cieśluk, Dorota Sadowska, Justyna Krzepota

**Affiliations:** 1 Institute of Physical Culture Sciences, University of Szczecin, al. Piastów 40B, Blok 6, 71-065 Szczecin, Poland; kacper.ciesluk@usz.edu.pl; 2 Department of Physiology, Institute for Sport—National Research Institute, ul. Trylogii 2/16, 01-982 Warsaw, Poland; dorota.sadowska@insp.waw.pl

**Keywords:** physical exercise, FITLIGHT Trainer^TM^, youth, training, reaction time, RAMP warm-up

## Abstract

The assessment of the various skills of athletes is carried out in terms of their ability to perform sport-specific tasks. The cognitive abilities of the players have significance for their effectiveness. In volleyball, a player’s ability to react quickly appears to be crucial in responding to an opponent’s dynamic play. The aim of the study was to evaluate the changes in reaction time to light signals following warm-up and physical exercise. Eighteen volleyball players (15.58 ± 2.01 years) participated in the study. Four FITLIGHT Trainer^TM^ discs were placed on the wall facing the participant to conduct the reaction time test. The participant’s task was to react as quickly as possible to the illuminated disc by touching it with the palm of their hand. The procedure was repeated five times: before the warm-up, after the warm-up, and after each of the three agility tests. Friedman’s ANOVA showed statistically significant differences in reaction time between the tests (Chi^2^ ANOVA = 61.23; *p* < 0.001). All tests performed after the warm-up according to the RAMP protocol showed statistically significantly better results than those before the warm-up (*p* ≤ 0.05). At the same time, no differences were observed between the tests performed after successive agility tests. The results indicated that a well-chosen warm-up plays an important role in shortening the time of visual-motor reaction to a light stimulus (RT). Subsequent studies should be expanded to include other research groups and assess other parameters.

## 1. Introduction

Repeated modifications of volleyball regulations place high demands on the improvement of training methods and measures to adapt the training process to the development of the sport and to continuously improve the sports skill level of the players. It has been documented that visual function [1,2] and the visual search processes of tracking multiple moving objects simultaneously, anticipatory ability, and visual-motor reaction time play key roles in volleyball [3,4,5]. Special training can contribute significantly to their improvement [6,7]. The right decision and quick response to visual stimuli are important aspects of scientific research, especially in typically technical and tactical actions [8,9,10]. Although it is clear that warming up contributes to the player’s performance [11] and reduces the risk of injury, particularly among children and adolescents [12,13], finding the optimal type of stretching to meet the demands of volleyball remains a challenge. In this respect, the problem of the warm-up has been the focus of several studies [14,15]. The most frequently addressed research problem is the effect of different warm-up protocols on vertical jumps [14,16,17,18,19,20,21,22]. Furthermore, some authors [18] studied the effect of static stretching or active warm-up (isolated or combined) on the results of functional tests (the agility test (T-Drill) and 20 m sprint test), while others [23] evaluated the results of fitness tests following dynamic stretching, warm-up with a foam roller, and their combinations. Previous studies have also looked at the type of dynamic and combined dynamic-static warm-ups in relation to kinematic landing patterns for the dominant and non-dominant limb in search of factors to counteract landing injuries [24], as well as improving blocking agility and leg muscle power using 1-minute whole body vibration stimulation [25].

Our attention and curiosity were aroused by the problem of reaction time, which plays a key role in maintaining an athlete’s psychomotor performance, especially during increasing fatigue [26]. In modern volleyball, the game is characterized by high speed, and, consequently, shorter exchanges, which translates into a longer time when the ball is out of play [27]. Players must regularly and often switch from a short phase of maximum psychophysical effort to a longer phase of passive play [28]. Previous research dedicated to the evaluation of players’ reaction times has focused on, among other things, comparing adult [29] and youth [30] players specializing in playing in different positions. We failed to identify previous studies conducted to assess visual-motor reaction time immediately after warm-up and after the performance of short repeated sport-specific volleyball efforts by young players. Furthermore, Mroczek et al. [31,32], who conducted a study among elite volleyball players, observed that reaction times are reduced under match conditions. Recent reports have provided important clues for the training process in terms of reducing the reaction time of volleyball players [33,34,35,36,37,38], suggesting the usefulness of Fitlight technology [33,34,35]. Therefore, the aim of the study was to evaluate changes in the time of reaction to light signals following the RAMP warm-up and repeated short-term volleyball-specific physical exercise using Fitlight technology. We were interested in the effect of performing the warm-up according to the RAMP method, as it involves the use of exercises according to three phases: raise—low-intensity activity to raise body temperature; activate and mobilize—activate key muscle groups, mobilize key joints and ranges of motion used in the sport; and potentiate—activities of increased intensity similar to the efforts in the later part of training or game [39].

## 2. Material and Methods

### 2.1. Participants

Eighteen volleyball players participated in the study. The mean age of the study participants was 15.58 ± 2.01 years. The tests were conducted during a training session with the permission of the coach and the club president. All study participants and their legal guardians were informed about the study procedure. Written, informed, and voluntary consent to participate in the study was obtained from adults and from legal guardians in the case of minors.

### 2.2. Testing Procedure

Measurements were taken in two groups of nine. The first group took the test on one day and the second group on the following day. Both groups took the tests at the same time. Training started on a weekday at 18:00. The players arrived at the venue 15 min before the start of classes. Upon entering the sports hall, participants were introduced to the research procedure. Next, the measurement of the time of visual-motor reaction to a light stimulus (RT) (task before the warm-up) was started sequentially by each participant.

**Figure 1:**
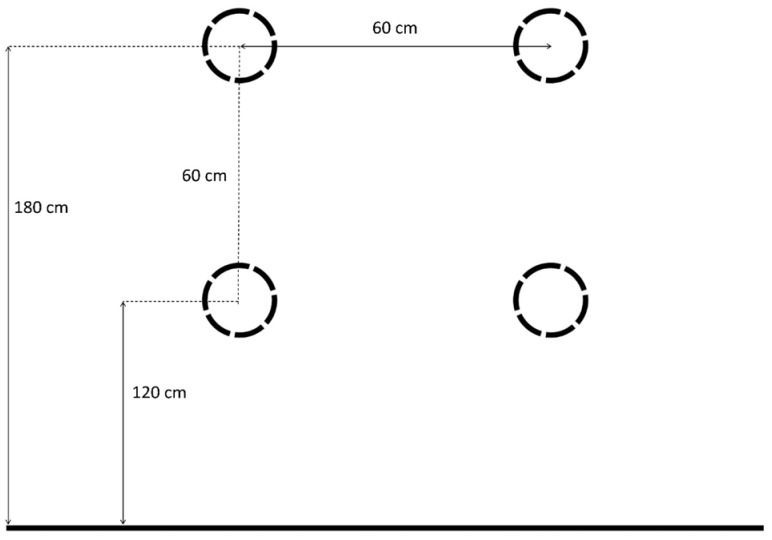
FITLIGHT Trainer^TM^ light disc arrangement.

Order of the RT task. The study used four discs of the FITLIGHT Trainer^TM^ (FITLIGHT^®^ Corp. Aurora, ON, Canada) system attached to the wall. The layout of the discs with the distance between them is presented in Figure 1.

**Figure 2:**
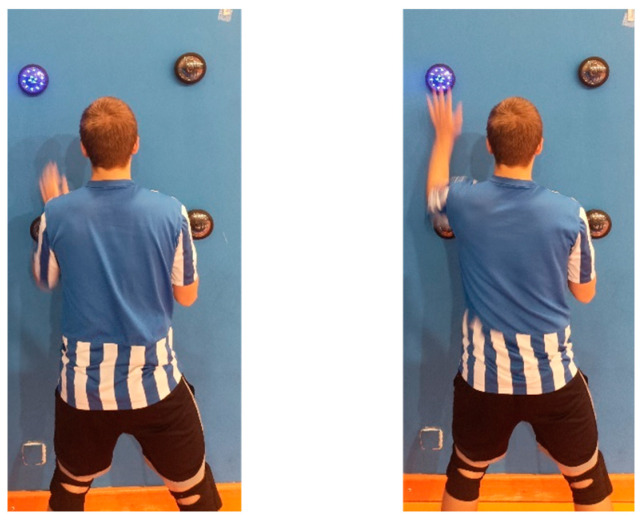
Player’s behaviour in response to a light signal.

The start of the task was preceded by three colours (red, yellow, and green) displayed consecutively on one of the discs. After the green colour went off, the actual task followed, which consisted of 16 reactions of the player to the blue light signal, four for each disc. There was a time interval of 0.7 s between when each disc was off and on (displayed). The order in which the discs were displayed was randomly generated (by a random number generator) before the study and set in the same way for each participant. The player facing the wall in a ready-to-block position had to respond as quickly as possible after the light signal was displayed, by touching it with the palm of their hand (Figure 2). Every reaction of the player was recorded and saved by the device in milliseconds. The test task took approximately 15–20 s to complete, depending on the player’s reaction time.

Once all participants had completed the task, the first person started the warm-up (15 min). Each person started their warm-up 10 min after the warm-up begun by the previous participant. From the beginning of the warm-up, the whole procedure took about 20 min.

The warm-up. The warm-up was performed using the RAMP protocol. It consisted of three elements: (a) Raise—increase body temperature; (b) Activate and Mobilise—activate individual muscle groups and mobilise them; (c) Potentiate—high-intensity dynamic exercises [39]. The warm-up time was 15 min and consisted of the elements shown below.

(a) Raise. Aerobic running exercises (3 min). Each player ran three laps around the volleyball court. After each lap, the player performed a move without the ball in each zone in the defensive line.
(b) Activate and Mobilise. Postural muscle activation exercises (5 min). Kneeling, in front support, and lying down, the athletes performed the raising and lowering of upper and lower limbs in different planes. Dynamic stretching (5 min). From different starting positions, the athletes performed arm swings and arm circles, trunk bends and twists, lunges and squats.
(c) Potentiate. Accelerations of several seconds (2 min). The athletes performed several sprints in the volleyball court area with dynamic changes of direction.

After completing the warm-up, the player immediately proceeded to the second RT measurement (the task after the warm-up). Next, after a minute’s break (passive rest), the player started the modified agility T-Test.

Modified agility T-Test. Agility test, including elements specific to the court tasks of volleyball players. For the purposes of the T-Test presented in the study [40], the test was modified to create additional volleyball-specific situations. The forward and backward running distance was extended to 10 m; then, instead of a shuffle to the cone, the players performed a move and block on either side of the court over the net in any way.

**Figure 3:**
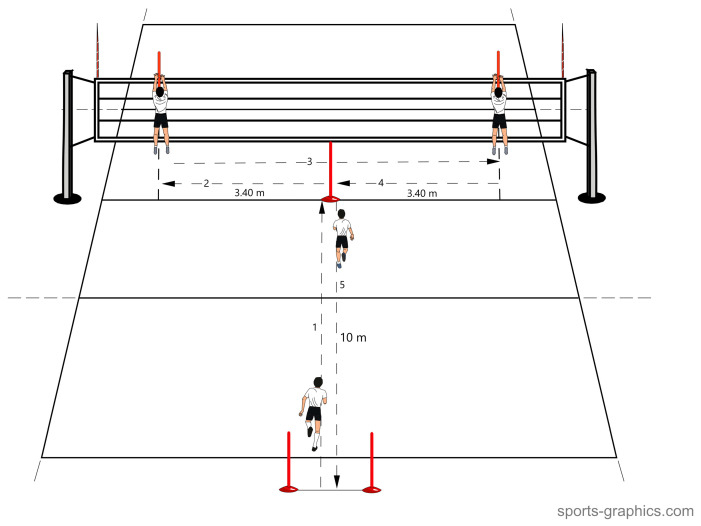
Example of performing the modified agility T-Test procedure by a player moving to the block first to the left and then to the right. After the first modified agility T-Test, the player immediately proceeded to the third RT (the task after the first exercise-modified agility T-Test). The player then repeated the entire procedure (modified agility T-Test and RT) twice. Each of the RT tasks differed in the order in which the discs were displayed to avoid a learning effect.

Each player, standing on the axis of the court and one meter behind the end line, ran to the net (10 m) and touched, with both hands, the pole set in the center of the court on the centerline. The player then moved to the right or left side of the block and executed the block in such a way that the pole set over the top edge of the net was between the player’s hands. After performing the block, the player returned to the pole set in the middle and performed another block to the other side. Then, the player returned to the pole set in the middle, touched it with both hands and, moving backward, returned to where he started the test (Figure 3). The modified agility T-Test took 10–20 s to complete, depending on the speed of the players.

**Figure 4:**
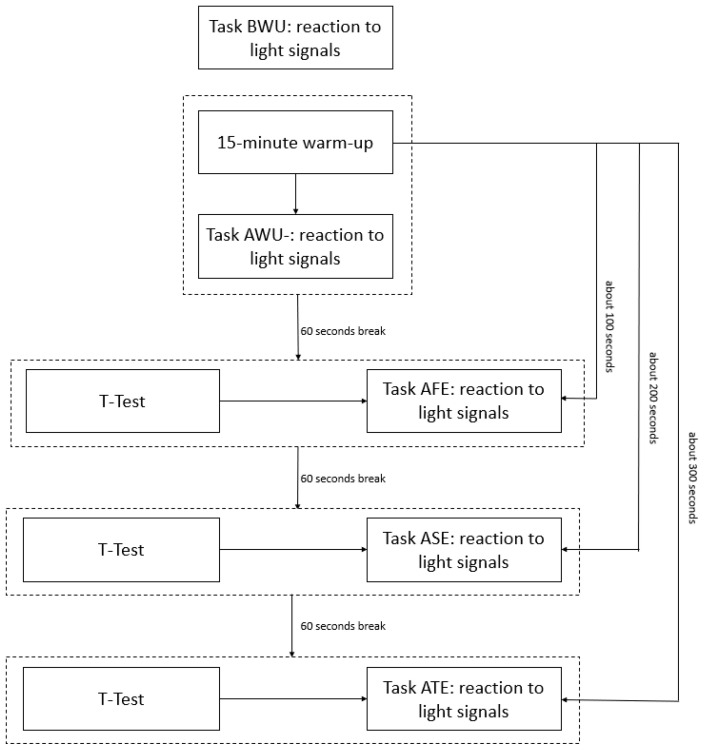
Diagram of the research procedure. BWU—before the warm-up; AWU—after the warm-up; AFE—after the first exercise modified agility T-Test; ASE—after the second exercise modified agility T-Test; ATE—after the third exercise modified agility T-Test.

Overall, the player performed the task five times: before the warm-up, after the warm-up, after the first exercise modified agility T-Test, after the second exercise modified agility T-Test, and after the third exercise modified agility T-Test (Figure 4).

### 2.3. Statistical Analysis

Statistical analysis was performed using the STATISTICA 13.1 software. The statistical significance level was set at *p* ≤ 0.05. Analyses with repeated measurements for the dependent groups were used to assess the significance of differences in reaction time measured at multiple points in time (before warm-up, after warm-up, after the 1st T-Test, after the 2nd T-Test, and after the 3rd T-Test). The calculations began by verifying that the assumptions of the parametric ANOVA test with repeated measures were met. The normality of distribution of individual variables was verified using the Shapiro–Wilk test. All distributions differed from the normal distribution. In addition, the variances between all combinations of related groups were not equal. Therefore, a non-parametric Friedman’s ANOVA test was used for the calculations. The post hoc analysis used Dunn’s test with a Bonferroni correction.

## 3. Results

Table 1 shows descriptive statistics for measurements collected before the warm-up, after the warm-up, and after each of the three exercise tests. Friedman’s ANOVA showed statistically significant differences between the tests ( Chi^2^ ANOVA = 61.23; *p* < 0.001) (Table 2). Post hoc analysis showed that the response time to the light signal was significantly longer before the warm-up compared to later measurements (*p* ≤ 0.05). No statistically significant differences were observed between the other measurements.

## 4. Discussion

The aim of the study was to assess changes in reaction time to light signals following the RAMP warm-up and repeated short-term volleyball-specific bouts of physical exercise. The results of the study showed that the time of players’ reaction to light signals shortened after the warm-up and remained at a similar level following subsequent short-term efforts. No significant differences were observed in reaction times measured between successive efforts performed after the warm-up. There are many studies in the literature focusing on the problems of reaction time changes in different sports, but there is still a lack of reports describing the effect of the warm-up on the time of visual-motor reaction of volleyball players. While some researchers have observed a reduction in reaction time, regardless of the type of warm-up [41,42,43], others have observed no change in reaction time between warm-up protocols [19,44,45,46].

The results of our study confirm the beneficial effect of warm-up on volleyball players’ reaction times. Interestingly, the study [47] found that the warm-up had a greater effect on recognition and reaction time for more difficult light stimuli. In contrast, in our study, we also noted its effect on a simple upper limb visual-motor task. A similar procedure to the one presented in this paper was used in a study of basketball players [41], where a reduction in visual reaction time after warm-up was also reported for both the dominant and non-dominant upper limbs, with no significant differences for the lower limbs. In our study, the task was performed by the player with both upper limbs. It is also worth noting that publications have highlighted the importance of using sport-specific exercises during the warm-up, which helped improve disjunctive reaction time among football players [42] and reaction time to an incoming ball among table tennis players [43]. Perhaps the explanation for our results should be sought in the RAMP warm-up protocol, in which dynamic exercises performed in the final phase are designed to stimulate the nervous system.

The importance of exercise for visual and cognitive processes and function in both adults [48] and children [49] and for reaction time has been documented in the literature [26,50]. Furthermore, our previous study [51] showed that repeated short-term physical exercise does not reduce the quality of volleyball movement tasks. Although the quality of the technical actions performed and even the speed of decision-making are dependent on the players’ sports skill level [52], it is the right decision and reaction time that can determine the further execution of a movement task, especially during defensive actions such as a block [8]. In our study, conducted under training conditions, we introduced three short-term volleyball-specific physical efforts (modified agility T-Test) after the warm-up to see how the time of visual-motor reaction to light stimuli changes under conditions of increasing fatigue. The findings presented by Mroczek et al. [31,53] indicated that elite volleyball players do not exceed the psychomotor fatigue threshold during a match, and their reaction time significantly decreases during the match compared to the pre-warm-up period. With regard to the results presented by the authors, our study seems to confirm that volleyball players, including young people, who are at a different level of athletic performance, maintain shorter times of visual-motor reaction after the warm-up and during subsequent short-term volleyball efforts.

However, our study has some limitations. First, we conducted one type of warm-up (used the established and accepted RAMP warm-up protocol) and did not make a comparison of what results we would get by conducting a different type of warm-up. Second, there was no control group in our study, which would have allowed us to draw more detailed conclusions. Therefore, we are aware that our conclusions would have to be approached with great caution because we cannot conclude whether the observed changes were induced by the RAMP warm-up protocol or whether they can occur after other types of warm-up. Third, we also did not assess any motor fitness elements of the volleyball players, as was done by [25,54,55]. Therefore, we do not know how the observed reduction in reaction time following the warm-up translates into the level of technical and tactical parameters. Moreover, the research included only a small group of male volleyball players at the initial stage of training. Extending the study to more advanced groups and increasing the number of participants, both women and men, could yield further interesting findings. Finally, we also realize that to provide a more complete picture of the characteristics of the player’s behavior, the research should, in the longer term, include measurements related to visual perceptual skills, attentiveness, anticipation, and decision-making.

## 5. Conclusions

The results of the study showed that reaction times to light signals significantly decreased in youth volleyball players after a warm-up following the RAMP protocol. The findings highlight the importance of an appropriately programmed warm-up, after which athletes improve their ability to respond to emerging visual stimuli.

## Figures and Tables

**Table 1:** Descriptive statistics for reaction time in milliseconds (ms) measurements taken before warm-up, after warm-up and after subsequent T-Tests.

	M	SD	Mdn	Min.	Max.	Q1	Q3
Before the warm-up	435.99	87.04	433.00	190.00	860.00	484.00	376.00
After the warm-up	400.47	66.81	394.00	188.00	660.00	430.00	354.00
After the 1st T-Test	395.43	67.33	386.00	245.00	738.00	434.00	349.00
After the 2nd T-Test	403.87	68.53	406.00	210.00	614.00	444.00	354.00
After the 3rd T-Test	397.26	71.38	394.00	238.00	880.00	438.00	348.00

M—mean; SD—standard deviation; Mdn—median; Min—minimum; Max—maximum; Q1—lower quartile; Q3—upper quartile.

**Table 2:** Significant differences for reaction time measurements taken before warm-up, after warm-up, and after subsequent T-Tests with post hoc analysis.

	Chi^2^ ANOVA	Before the Warm-Up	After the Warm-Up	After the 1st T-Test	After the 2nd T-Test	After the 3rd T-Test
Before the warm-up	61.23 *p* < 0.001	-	≤0.05	≤0.05	≤0.05	≤0.05
After the warm-up		≤0.05	-	ns	ns	ns
After the 1st T-Test		≤0.05	ns	-		ns
After the 2nd T-Test		≤0.05	ns	ns	-	ns
After the 3rd T-Test		≤0.05	ns	ns	ns	-

ns—not significant.

## Data Availability

The data presented in this study are available on request from the corresponding author.
